# Social Exclusion Down-Regulates Pain Empathy at the Late Stage of Empathic Responses: Electrophysiological Evidence

**DOI:** 10.3389/fnhum.2021.634714

**Published:** 2021-03-01

**Authors:** Min Fan, Jing Jie, Pinchao Luo, Yu Pang, Danna Xu, Gaowen Yu, Shaochen Zhao, Wei Chen, Xifu Zheng

**Affiliations:** ^1^Key Laboratory of Brain, Cognition and Education Sciences (South China Normal University), Ministry of Education, Guangzhou, China; ^2^Guangdong Key Laboratory of Mental Health and Cognitive Science, School of Psychology, Center for Studies of Psychological Application, Guangdong Key Laboratory of Mental Health and Cognitive Science, South China Normal University, Guangzhou, China; ^3^School of Biomedical Engineering, Hainan University, Haikou, China; ^4^School of Education and Psychological Science, Sichuan University of Science, and Engineering, Zigong, China; ^5^School of Management, Guangzhou Vocational College of Science and Technology, Guangzhou, China; ^6^China People's Police University, Guangzhou, China

**Keywords:** pain empathy, social exclusión, ERP, N2, P3, LPP

## Abstract

Social exclusion has a significant impact on cognition, emotion, and behavior. Some behavioral studies investigated how social exclusion affects pain empathy. Conclusions were inconsistent, and there is a lack of clarity in identifying which component of pain empathy is more likely to be affected. To investigate these issues, we used a Cyberball task to manipulate feelings of social exclusion. Two groups (social exclusion and social inclusion) participated in the same pain empathy task while we recorded event-related potentials (ERP) when participants viewed static images of body parts in painful and neutral situations. The results showed early N2 differentiation between painful and neutral pictures in the central regions in both groups. The pattern at the late controlled processing stage was different. Parietal P3 amplitudes for painful pictures were significantly smaller than those for neutral pictures in the social exclusion group; they did not differ in the social inclusion group. We observed a parietal late positive potential (LPP) differentiation between painful and neutral pictures in both groups. LPP amplitudes were significantly smaller in the social exclusion group than those in the social inclusion group for painful stimuli. Our results indicate that social exclusion does not affect empathic responses during the early emotional sharing stage. However, it down-regulates empathic responses at the late cognitive controlled stage, and this modulation is attenuated gradually. The current study provides neuroscientific evidence of how social exclusion dynamically influences pain empathy.

## Introduction

Empathy, the natural ability to share and understand the emotions of others while being aware of the distinction between the self and others (Decety and Jackson, 2004; Singer and Lamm, 2009; Luo et al., 2012; Coll et al., 2017), has great significance in our social life. Empathy gives us important information about other people and the environment, making it more possible to handle potential threat and promote prosocial behavior (Frith and Frith, 2006; Decety, 2010; Graaff et al., 2018). Several studies revealed that empathy is influenced by contextual factors, such as fairness (Singer et al., 2006), social distance (Meyer et al., 2012; Wang et al., 2016), competition (Yamada et al., 2011; Luo et al., 2018a), state anxiety (Luo et al., 2018b), self-interest (Jie et al., 2019a,b), as well as social exclusion.

It is well-established that people have a fundamental need to belong (Baumeister and Leary, 1995; Williams, 2007). What happens when this need to belong is threatened, a situation most people experience from time to time (Williams, 2009)? A large number of studies have suggested that social exclusion has significant effects on cognition (Twenge et al., 2003; Baumeister et al., 2005), emotion (Blackhart et al., 2009; Gerber and Wheeler, 2009) and behavior (Twenge et al., 2001, 2002; Leary et al., 2003; Guerra et al., 2004; DeWall et al., 2010; Masten et al., 2011).

Studies have explored how social exclusion influences empathy. Some studies have observed that receiving an ostensibly diagnostic forecast of a lonesome future life reduces subsequent empathic concern for a romantic breakup or a broken leg (DeWall and Baumeister, 2006; Cordaro, 2011). Another study adopting the same future-alone exclusion paradigm has found the intermediary role of a reduced level of empathic concern for a romantic breakup between social exclusion and a subsequent decline in prosocial behavior (Twenge et al., 2007). However, these findings contradict what other studies have found. It has been shown that the acute social exclusion induced by the Cyberball paradigm has no influence on empathy for an incurably sick sibling or a romantic breakup (Bass et al., 2014). Being socially excluded by Cyberball game even increases sensitivity to the social pain of others (Nordgren et al., 2011). Other researchers used functional magnetic resonance imaging (fMRI) to study the effect of social exclusion on the empathic response to social stimuli. Powers et al. (2011) discovered that in comparison with the social inclusion group, the Cyberball exclusion group did not show brain activation in regions involved in mentalizing and empathizing with others when watching negative social stimuli. Another fMRI study found the opposite results by showing that watching emotional social stimuli elicited stronger brain activation related to empathy after suffering Cyberball exclusion (Beyer et al., 2014).

In reviewing previous studies, it is suggested that there is still no consensus on the effect of social exclusion on pain empathy (DeWall and Baumeister, 2006; Cordaro, 2011; Bass et al., 2014). This inconsistent evidence might be associated with the differences between social exclusion paradigms. Research has shown that future-life exclusion brings about more severe injury and causes reduced physical pain sensitivity (namely pain tolerance and pain threshold), whereas Cyberball exclusion leads to less severe injury and causes hypersensitivity (Bernstein and Claypool, 2012). Reduced pain sensitivity also proved to mediate the negative role of future-life exclusion on pain empathy (DeWall and Baumeister, 2006). However, no research has explored how hypersensitivity induced by Cyberball exclusion affects pain empathy. Another explanation may be related to the measurement of empathy. Bass et al. (2014) mentioned that different empathic responses to social exclusion might be attributed to the way we measure empathy. Most studies adopted self-reported empathy, which might easily be affected by various factors that could influence responses, for example, social desirability (Deshields et al., 1995; Logan et al., 2008; Cordaro, 2011). Only two relevant fMRI studies investigated neural activity but they did not study the specific field of pain empathy, and the results were contradictory (Powers et al., 2011; Beyer et al., 2014). Also, there are indications in recent event-related brain potential research that pain empathy is a dynamic process involving an early automatic affective arousal component (N1/N2) and a late controlled cognitive evaluation component (P3/LPP) (Fan and Han, 2008; Decety et al., 2010; Wang et al., 2016; Luo et al., 2018b). It remains to be elucidated how acute social exclusion modulates pain empathy, and at what phase of information processing does this regulation occur. ERP is widely used in affective neuroscience research due to its high temporal resolution compared to fMRI (Fan and Han, 2008; Decety et al., 2010). Meanwhile, there is evidence that ERP is extensively applied in the field of lie detection (Farwell and Donchin, 1991) because it is less susceptible to social desirability (Mostafa, 2014). Therefore, we adopted ERP to compare the empathic response in participants from a social exclusion group and a social inclusion group when they were viewing static images of body parts showing painful and neutral situations. It has been demonstrated that playing an online ball-tossing game, Cyberball, can induce feelings of social exclusion (Williams et al., 2000; Zadro et al., 2004, 2006; Williams and Jarvis, 2006). Here, we asked participants to play the same ball-tossing game, the only difference between groups being whether they could play with other players during the entire process.

Recent ERP studies have shown that pain empathy response is a dynamic process indexed by the differentiation between pain and no-pain. This process contains an early automatic affective sharing component (N1/N2) and a late controlled cognitive evaluation component (P3/LPP) (Fan and Han, 2008; Decety et al., 2010; Coll, 2018a; Luo et al., 2018b). Previous studies have found that painful stimuli elicit a more positive N2 shift relative to neutral stimuli. An ERP study investigating the effect of physical pain on pain empathy suggests that physical pain only affects late cognitive evaluation during pain empathy. Therefore, we assumed that, as a form of pain, social exclusion would not affect early empathic responses, whereby, regardless of group, participants would exhibit a more positive shift in N2 amplitudes when watching painful stimuli in contrast with neutral stimuli. The late empathic component P3/LPP is supposed to be driven by top-down control. Previous studies found that high levels of executive functions, including self-control and response inhibition, are important for top-down controlled empathic processes (Decety and Jackson, 2004; Decety and Lamm, 2006; Mella et al., 2012). Social exclusion has been found to impair self-control (Baumeister et al., 2005; Campbell et al., 2006; DeWall et al., 2008) and response inhibition (Otten and Jonas, 2012; Xu et al., 2016), while social inclusion has been shown to promote self-regulation (DeWall et al., 2008) and cognitive functioning (Shapira et al., 2007). Meanwhile, it has been shown that P3/LPP is more positive in response to painful stimuli than to neutral stimuli (Fan and Han, 2008; Coll, 2018a). Therefore, we hypothesized that painful stimuli would evoke a larger P3/LPP relative to neutral stimuli. Moreover, we predicted that P3/LPP differences between painful/neutral stimuli might be reduced or even not observed in the social exclusion group but not in the social inclusion group because social exclusion might impair participants' late top-down controlled processing of others' pain. Then it might hinder the late empathic responses. However, social inclusion could have the opposite impact. We explored the results of behavioral empathy assessments such as self-unpleasantness and other-unpleasantness scores in behavioral pain empathy tasks.

## Materials and Methods

### Participants

Forty-five college students participated in this study as paid volunteers. Three of the subjects (three females) were excluded from data analysis because of excessive artifacts during the EEG recording. The behavioral and EEG data were reported from forty-two subjects (23 females, mean age 19.79 ± 5.96 years). There were twenty-two participants (12 females) in the social exclusion group and twenty participants (11 females) in the social inclusion group. As for justification for the chosen sample size, we performed a power analysis by G^*^power3.1(Faul et al., 2007). In order to detect medium effect sizes (Cohen's *f* = 0.25) with 80% power, a minimum of 34 participants were required. All subjects were right-handed, with normal or corrected-to-normal vision and had no neurological or psychiatric history (in the participants recruitment information we have listed these requirements and we have asked participants to give relevant oral report after they came to the lab). Before the study commenced, informed consent was obtained from each subject. The study was approved by the Academic Committee of South China Normal University. The experimental procedure met the standard of ethical standards of the Declaration of Helsinki (British Medical Journal Publishing Group, 1996).

### Materials

We adopted 60 digital color photographs similar to those used in previous ERP studies (Fan and Han, 2008; Luo et al., 2018a) in the empathy task during the ERP session. The visual stimuli comprised 30 pictures showing hands in painful situations and 30 matching pictures showing hands in neutral situations. The painful pictures showed situations such as a hand trapped in a door or cut by scissors. Each 10.4 × 7.5 cm (width × height) picture was presented in the center of a 17-in. color monitor against a white background.

### Procedure

After signing the informed consent, participants were instructed to play a Cyberball game with other two computerized players, ostensibly with other participants who stayed in another lab, during which they were told to mentally visualize the whole scene (Williams et al., 2000). We manipulated this task so that participants randomly assigned to the social exclusion group caught the ball three out of 30 throws, and those randomly assigned to the social inclusion group caught the ball about ten times. The Cyberball game last for 4–5 min. Immediately following the Cyberball game, each participant was required to rate the self-reported social exclusion and social ostracism scores on a 5-point scale (1 = no exclusion, 5 = extremely high exclusion, or 1 = no ostracism, 5 = extremely high ostracism). To ensure that this manipulation induced feelings of being socially excluded continued through the experiment, participants were asked to complete the rating task again after the experiment. After the rating task, participants filled in a self-reported levels of needs questionnaire with four subscales: belonging, control, self-esteem, and meaningful existence (Zadro et al., 2004) and the Positive and Negative Affect Scale (PNANS) containing 20 items assessing positive emotions and negative emotions (10 each) (Watson et al., 1988).

Immediately after the measurement of those questionnaires, the pain empathy task starts, which lasted for 18–22 min. In the ERP session, all participants performed the same pain empathy task while event-related brain potentials were recorded. Participants were instructed to judge pain vs. no-pain for hands in painful and neutral pictures. There were 200 trials, of which 20 trials were for practice. ERP recordings consisted of four blocks, each containing 45 trials, and the stimuli in each block of trials were presented in random order. Each trial began with a red fixation cross with a duration ranging from 500 ms to 800 ms, followed by a picture for 1,000 ms. Then, after a break of 1,500–2,000 ms, a question mark remained for up to 3,000 ms before a response was given and the subjects needed to recognize the content of the stimuli (painful or neutral) by pressing a button. Finally, a white screen appeared for 500–800 ms. After the ERP recording session, participants were required to assess the unpleasantness experienced by people in the pictures (other) and by themselves (self) on a 5-point scale (1 = no unpleasantness, 5 = very unpleasant). Cyberball game and pain empathy task were programmed by E-prime 2.0.

At the end of the experiment, in order to measure individual differences in rejection sensitivity and trait empathy, subjects were asked to complete the Rejection Sensitivity Questionnaire (Feldman and Downey, 1994) and the Interpersonal Reactivity Index (IRI) (Davis, 1983).

### EEG Acquisition and Data Analysis

Electroencephalogram (EEG) data were recorded from 64 scalp electrodes mounted on an elastic cap according to the extended 10-10 system, with references on the left and right mastoids and AFz which stands vertically between electrode Fpz and Fz as the ground electrode. Eye blinks and vertical eye movements were monitored with electrodes located above the right eye. The EEG activity was amplified at 0.01–100 Hz band-passes and sampled at 500 Hz. All electrode impedance were kept below 5 kΩ. ERPs under each condition were computed separately off-line using Brain Vision Analyzer 2.0 software (Fritsch and Kuchinke, 2013). EEG data recording, pre-processing and analysis were performed by Brain Products, Germany. ERPs at each electrode were re-referenced to the algebraically computed average of the left and right mastoids before further analysis. We set the high cutoff value of filter to be 30 Hz. The data under each condition were averaged separately off-line, and each epoch continued for 1,200 ms with 200 ms before the picture onset for baseline correction. Trials contaminated by eye blinks, eye movements and muscle potentials exceeding 100 mV at any electrode or response errors were excluded from the average. 7.43% of the trials were excluded due to artifacts (social exclusion group: painful pictures = 6.87%; neutral pictures = 7.58%; social inclusion group: painful pictures = 7.56%; neutral pictures = 7.61%). In the social exclusion group, the effective number of trials for painful pictures and neutral pictures were 83.82 ± 10.23 and 83.18 ± 11.03, respectively. Similarly, in the social inclusion group, the effective number of trials for painful pictures and neutral pictures were 83.05 ± 11.74, 83.15 ± 12.11, respectively. Trials with the incorrect response for pain categorization were still included in this process and in later data analysis.

According to the results of previous ERP studies (Fan and Han, 2008; Decety et al., 2010; Luo et al., 2018a,b) and the inspection of the grand-average data, we chose three ERP components: N2 (220–250 ms), a later component P3 (300–400 ms), and a late component LPP (450–750 ms). Previous meta-analysis studies on pain empathy found that the effect of pain observation on the N2 component is maximal at the frontal sites and most studies chose frontal-central region as the analyzed electrodes, and the effects of pain observation on the P3 and LPP components are maximal at the centro-parietal sites (Coll, 2018a,b). In the current study, for N2 component, we observed the largest effect of pain observation at the central sites. For P3 component and LPP component, we observed the largest effect of pain observation at the parietal sites. The selection of electrodes in each region was based on previous studies (Luo et al., 2018a,b). In sight of the results of the meta-analysis studies and the largest differences of pain observation through the inspection of the grand-average data, we included central (Cz, C3, C4) regions for N2 analysis, parietal (Pz, P3, P4) regions for P3 and LPP analysis. We averaged the electrodes for each region of interest to obtain the mean amplitudes for N2, P3, and LPP.

Spss17.0 was used to analyse behavioral and ERP data. To examine the effectiveness of social exclusion manipulation, independent sample *t*-test was conducted on self-reported social exclusion and social ostracism scores, need questionnaires sores as well as PANAS scores. A 2 (group: social exclusion group and social inclusion group) × 2 (stimuli: painful and neutral pictures) mixed ANOVAS was conducted on the subjective unpleasantness scores to investigate whether there exist group differences in behavioral pain empathy assessment. We also conducted a similar ANOVA on the reaction time and response accuracy of pain categorization in pain empathy task during EEG recording. A two-way mixed ANOVAS was performed for mean ERP amplitudes of each component for each region of interest with group (social exclusion, social inclusion) as a between-group factor, stimuli (painful pictures, neutral pictures) as a within subject factor. To investigate whether the electrophysiological activity was consistent with the subjective ratings of unpleasantness, Spearman correlation analysis was used to calculate the correlation between self-reported unpleasantness scores and the mean amplitudes of ERPs induced by painful pictures in each time window (Spearman correlations are usually more robust when correlating self-report and physiological data) (see Rousselet and Pernet, 2012).

Statistical differences were considered significant at *p* < 0.05 and adjusted Bonferroni correction was applied for pairwise comparisons. Homogeneity of variance of dependent variables in mixed ANOVAS models were examined and all the results showed homogeneity of variance.

## Results

### Behavioral Results

Table 1 presents the descriptive statistics for each subscale of the IRI, IRI total scores and RSQ. The results showed that no differences in PT, FS, EC, PD subscales, IRI total scores and RSQ scores were found between the social exclusion group and the social inclusion group (*p*s > 0.05).

**Table 1:** Mean scores and standard deviation for IRI and RSQ.

	**Social exclusion group**		**Social inclusion group**		***p*-value**
	**Mean**	**SD**	**Mean**	**SD**	
IRI total scores	51.22	9.23	52.00	8.77	*p* = 0.783
IRI-FS	15.55	3.57	15.50	3.24	*p* = 0.966
IRI-PT	12.55	3.29	10.85	3.05	*p* = 0.092
IRI-PD	7.27	4.15	7.75	4.15	*p* = 0.733
IRI-EC	15.86	4.23	17.9	1.88	*p* = 0.079
RSQ	9.76	2.59	11.24	3.19	*p* = 0.106

*FS, fantasy; PT, perspective taking; PD, personal distress; EC, empathic concern; IRI, Interpersonal Reactivity Index; RSQ, Rejection Sensitivity Questionnaire*.

**Figure 1:**
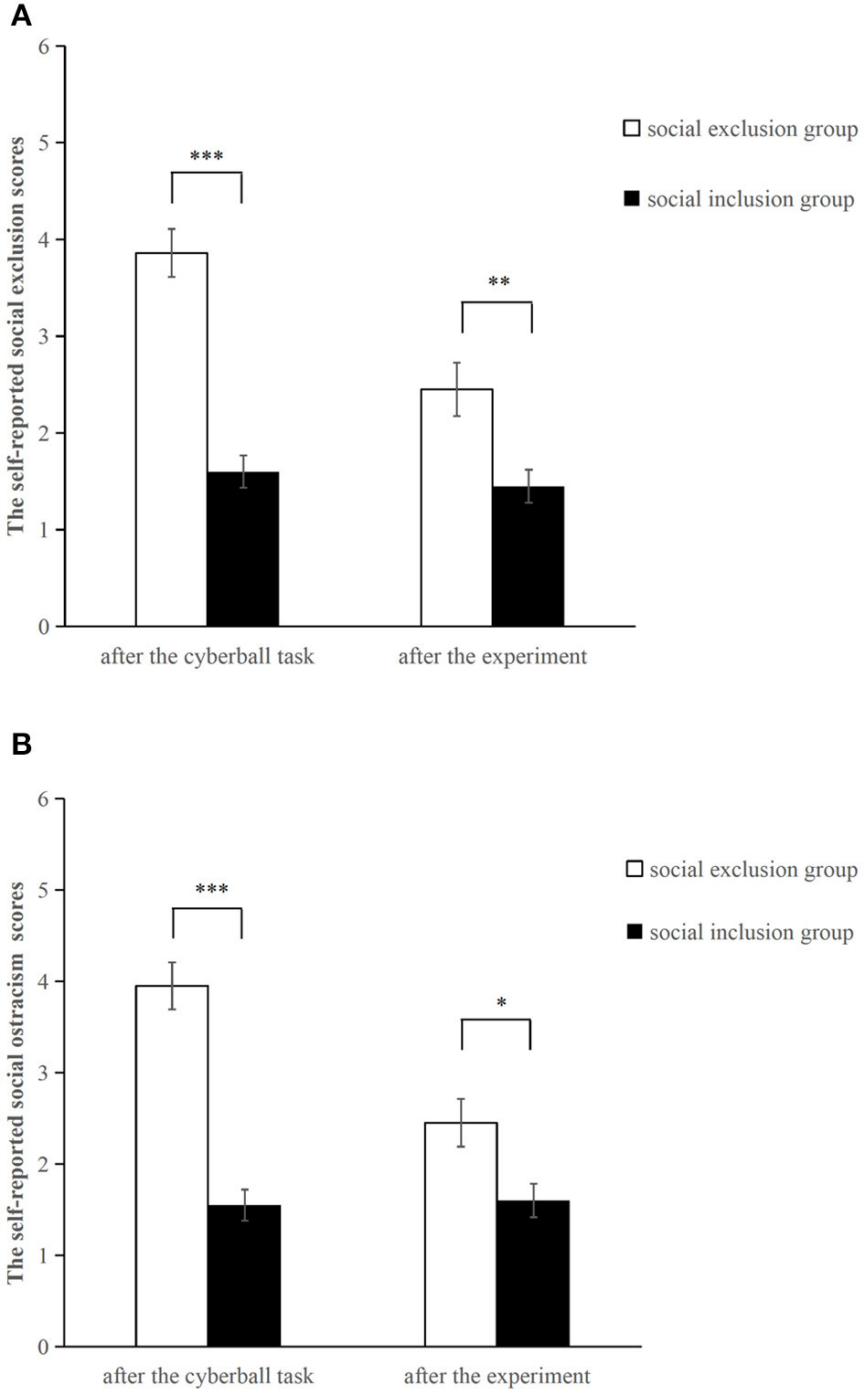
Self-reported social exclusion scores **(A)** and social ostracism scores **(B)** after the Cyberball task and after the experiment. *p < 0.05, **p < 0.01, ***p<0.001. Error bars denote standard errors.

An independent sample *t*-test was conducted to compare the self-reported social exclusion and social ostracism scores (see Figure 1). As expected, both scores in the social exclusion group were significantly higher than those in the social inclusion group after the Cyberball game (social exclusion scores: *t* = 7.383, *p* < 0.001, *d* = 2.3; social ostracism scores: *t* = 7.604, *p* < 0.001, *d* = 2.503). Such reliable differences continued up to the end of the experiment (social exclusion scores: *t* = 3.092, *p* = 0.004, *d* = 0.943; social ostracism scores: *t* = 2.679, *p* = 0.011, *d* = 0.816). The results revealed that the manipulation of the social exclusion was effective.

We also performed an independent sample *t*-test on the need questionnaires and the PANAS scores. The results showed that scores for belonging, control, self-esteem, and meaningful existence of the social exclusion group were significantly lower than those of the social inclusion group (*p*s < 0.001). The positive affect score of the social exclusion group was also significantly lower than that of the social inclusion group (*t* = −4.020, *p* < 0.001, *d* = 2.249), while the negative affect score of the social exclusion group was significantly higher than that of the social inclusion group (*t* = 4.338, *p* < 0.001, *d* = 1.353).

Finally, a two (group: social exclusion group and social inclusion group) ×2 (stimuli: painful and neutral pictures) mixed ANOVAS was conducted on the subjective unpleasantness scores. The results showed that the main effect of stimuli was significant in self-unpleasantness [*F*_(1,40)_ = 372.458, *p* < 0.001, η_*p*_^2^ = 0.903] and in other-unpleasantness scores [*F*_(1,40)_ = 507.693, *p* < 0.001, η_*p*_^2^ = 0.927]. Participants rated painful pictures as more unpleasant than neutral pictures from both perspectives (*p*s < 0.001). No other differences were found (lowest *p* = 0.581). We also conducted a similar mixed ANOVAS on reaction time and response accuracy of pain categorization. The results showed a significant main effect of stimuli on reaction time [*F*_(1,40)_ = 9.488, *p* = 0.004, η_*p*_^2^ = 0.192]. Painful pictures resulted in longer reaction time compared to neutral stimuli (*p* = 0.004). No other differences were found (lowest *p* = 0.296). The descriptive statistics of reaction time and response accuracy are presented in Table 2.

**Table 2:** The descriptive statistics for reaction time and response accuracy in pictures classification task (M ± SD).

**Group**	**Painful pictures**		**Neutral pictures**	
	**Reaction time (ms)**	**Accuracy (%)**	**Reaction time (ms)**	**Accuracy (%)**
**Social exclusion**	648.42 ± 39.81	94.14	605.16 ± 32.21	94.85
**Social inclusion**	588.53 ± 41.76	95.06	556.85 ± 33.78	95.50

### ERP Results

**Figure 2:**
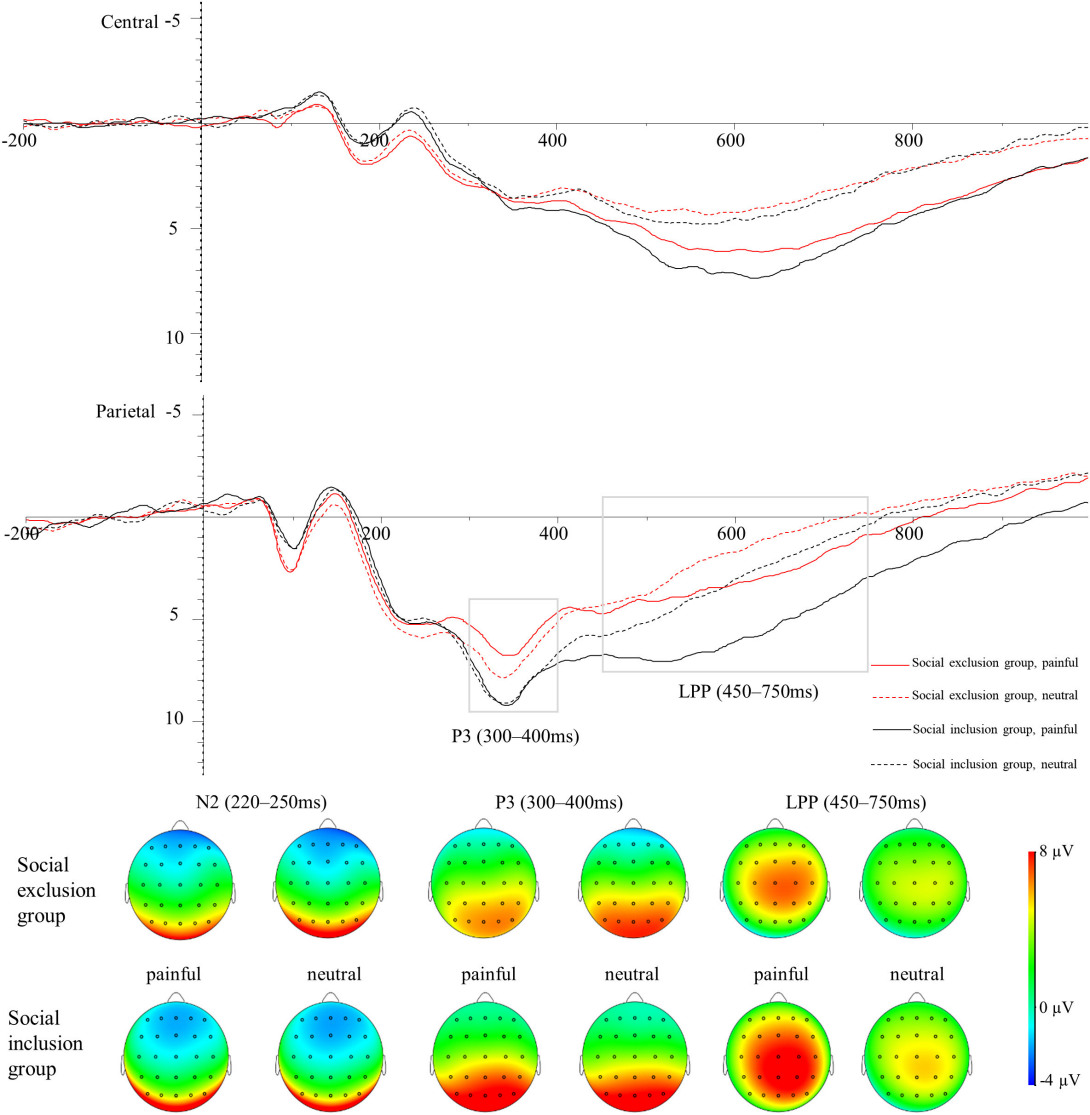
Average ERPs in the central, and parietal regions for painful pictures and neutral pictures in both groups. The voltage topographies illustrate the scalp distribution of N2, P3, and LPP components.

**Figure 3:**
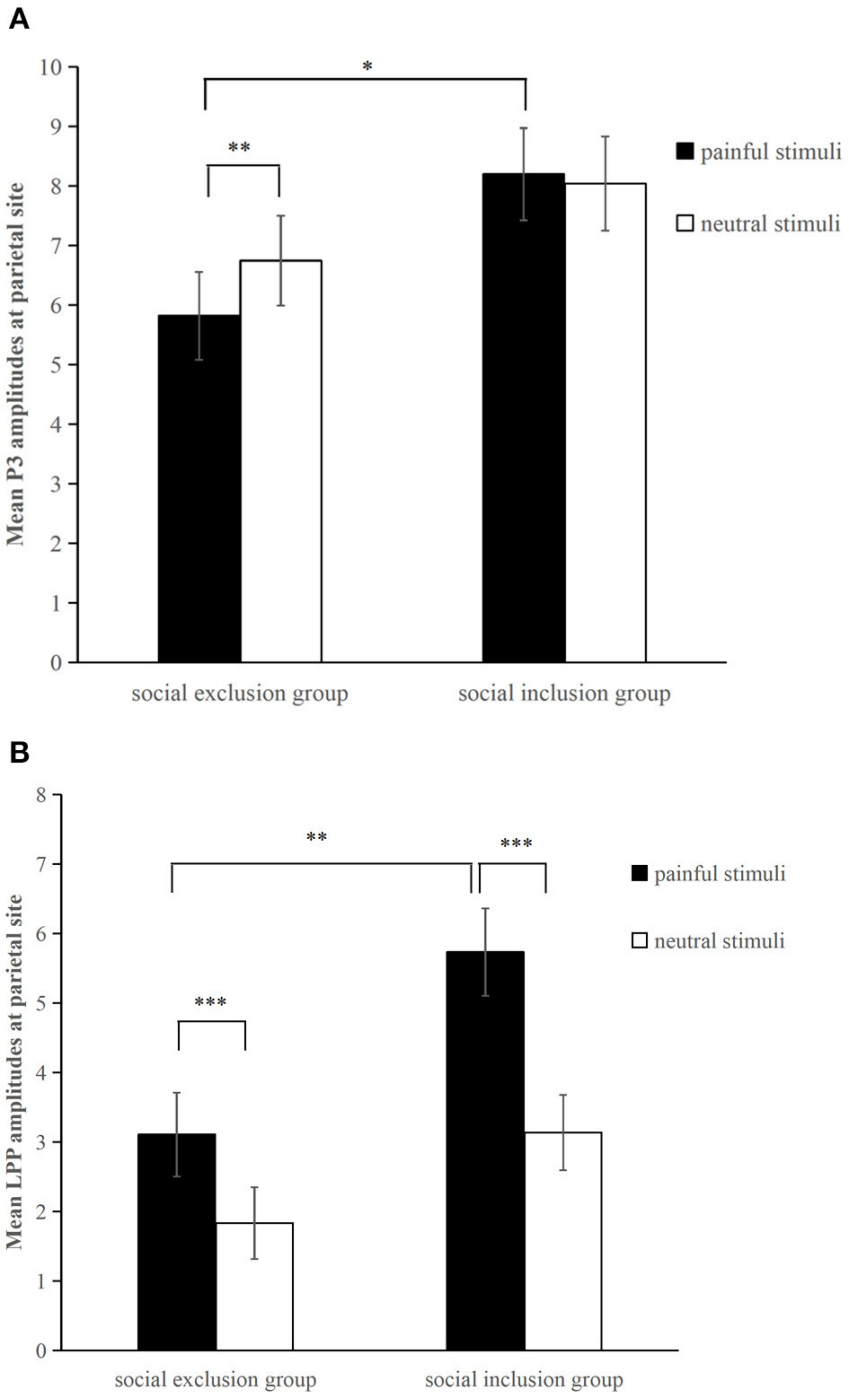
Averaged parietal (P3, P4, Pz) amplitudes for painful pictures and neutral pictures within the **(A)** P3 (300–400 ms) and **(B)** LPP (450–750 ms) time window in each group. *p < 0.05, **p < 0.01, ***p<0.001. Error bars denote standard errors.

**Figure 4:**
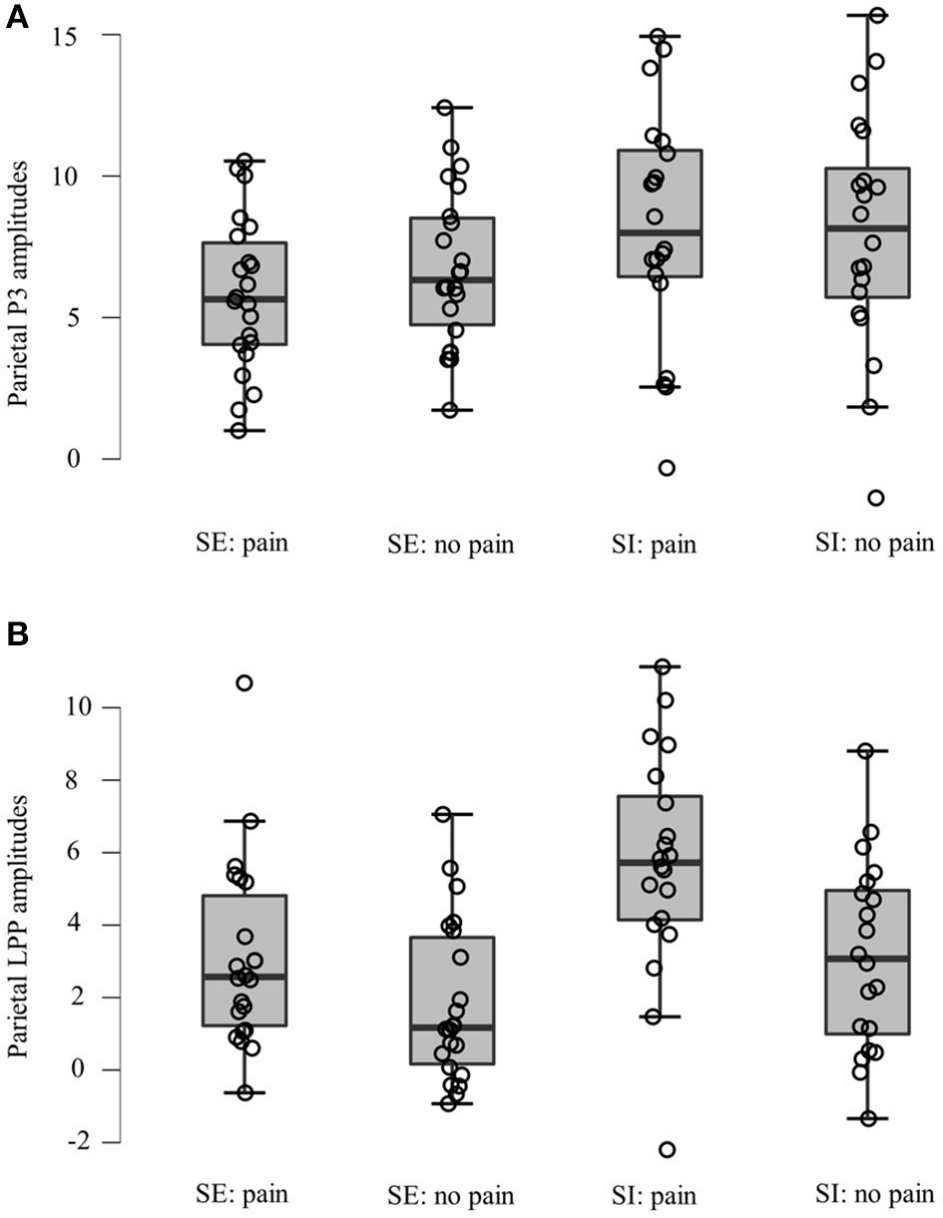
Data distribution of two groups in averaged parietal **(A)** P3 and **(B)** LPP amplitudes for painful pictures and neutral pictures. SE, social exclusion group; SI, social inclusion group.

The averaged ERPs at central and parietal regions and the voltage topographies are presented in Figure 2. Averaged parietal amplitudes within the P3 and LPP time window are illustrated in Figure 3. Figure 4 present data distribution of two groups in averaged parietal P3 and LPP amplitudes for painful pictures and neutral pictures.

For the N2 component, a 2 (group: the social exclusion group and the social inclusion group) ×2 (stimuli: painful and neutral pictures) mixed ANOVAS was conducted. There was a marginally significant main effect of stimuli [*F*_(1,40)_ = 4.094, *p* = 0.050, η_*p*_^2^ = 0.093] while the main effect of group was not significant [*F*_(1,40)_ = 2.808, *p* = 0.102]. Both groups exhibited a more positive shift in N2 amplitudes when watching painful stimuli in contrast with neutral stimuli (*p* = 0.05). The interaction between group and stimuli was not significant (*p* = 0.730).

For the P3 component, a two (group: the social exclusion group and the social inclusion group) ×2 (stimuli: painful and neutral pictures) mixed ANOVAS was conducted. The results showed a significant main effect of stimuli [*F*_(1,40)_ = 4.176, *p* = 0.048, η_*p*_^2^ = 0.095], while the main effect of group [*F*_(1,40)_ = 2.978, *p* = 0.092] was not significant. The interaction between stimuli and group [*F*_(1,40)_ = 8.23, *p* = 0.007, η_*p*_^2^ = 0.171] was significant. There was a reliable simple effect of stimuli in the social exclusion group [*F*_(1,40)_ = 12.669, *p* < 0.001, η_*p*_^2^ = 0.241] with painful stimuli eliciting smaller P3 amplitudes than neutral stimuli did. This effect was not significant in the social inclusion group [*F*_(1,40)_ = 0.325, *p* = 0.572]. In addition, simple effects of group were found significant under the pain condition [*F*_(1,40)_ = 4.934, *p* = 0.032, η_*p*_^2^ = 0.11] but not under non-pain condition [*F*_(1,40)_ = 1.411, *p* = 0.242]. Subsequent pairwise comparison showed that painful pictures induced significantly smaller P3 amplitudes in the social exclusion group than those in the social inclusion group (*p* = 0.032).

We conducted a similar ANOVAS on the average LPP amplitudes at the parietal site as we did on the average P3 amplitudes and the results showed a significant main effect of stimuli [*F*_(1,40)_ = 78.817, *p* < 0.001, η_*p*_^2^ = 0.663], and group [*F*_(1,40)_ = 6.291, *p* = 0.016, η_*p*_^2^ = 0.136]. There was a reliable two-way interaction between group and stimuli [*F*_(1,40)_ = 9.196, *p* = 0.004, η_*p*_^2^ = 0.187]. A significant simple effect of stimuli was observed in the social exclusion group [*F*_(1,40)_ = 17.939, *p* < 0.001, η_*p*_^2^ = 0.31] and in the social inclusion group [*F*_(1,40)_ = 67.704, *p* < 0.001, η_*p*_^2^ = 0.629] with painful stimuli eliciting larger LPP amplitudes than those elicited by neutral stimuli in both groups (*p*s < 0.001). There was also a reliable simple effect of group under the pain condition [*F*_(1,40)_ = 9.072, *p* = 0.004, η_*p*_^2^ = 0.185] but not under the neutral condition [*F*_(1,40)_ = 3.024, *p* = 0.09]. Subsequent pairwise comparisons suggested that the LPP amplitudes elicited in the social exclusion group were significantly smaller than those elicited in the social inclusion group (*p* = 0.004).

### Correlation Between Subjective Ratings and ERP Amplitudes

We also calculated the correlation between self-reported unpleasantness scores and the mean amplitudes of ERPs induced by painful pictures in each time window to explore if the subjective ratings of unpleasantness were correlated with the electrophysiological activity elicited by the painful pictures. Subjective other-unpleasantness ratings were significantly correlated with central N2 amplitudes for painful stimuli (*r* = –0.318, *p* = 0.04). The correlation between self-unpleasantness scores and central N2 amplitudes for painful stimuli were marginally significant (*r* = –0.291, *p* = 0.062). Subjective other-unpleasantness ratings were significantly correlated with parietal LPP amplitudes for painful stimuli (*r* = 0.311, *p* = 0.045) and marginally significantly. correlated with parietal P3 amplitudes for painful stimuli (*r* = 0.308, *p* = 0.050). However, after we corrected the alpha level for multiple comparisons by FDR (false discovery rate) we found none of these correlations were significant (lowest *p* = 0.093). Moreover, we also examine whether the group differences exist in these correlations. The LPP amplitudes induced by painful pictures in the parietal region were positively correlated with the subjective ratings of self-unpleasantness and other-unpleasantness in the social inclusion group (self-unpleasantness: *r* = 0.491, *p* = 0.028; other-unpleasantness: *r* = 0.478, *p* = 0.033) whereas no correlation was found in the social exclusion group (self-unpleasantness: *r* = −0.155, *p* = 0.49; other-unpleasantness: *r* = −0.038, *p* = 0.867). The P3 amplitudes induced by painful pictures in the parietal region were positively correlated with the subjective self-unpleasantness scores in the social inclusion group (*r* = 0.461, *p* = 0.041) whereas no correlation was found in the social exclusion group (*r* = −0.009, *p* = 0.968). However, the FDR results also showed that all the corrected *p* value in the social inclusion group were not significant (lowest *p* = 0.082). Therefore, our results showed that the ERP empathic responses are not significant with behavioral self-reported pain empathy.

## Discussion

Previous studies have investigated how social exclusion affects pain empathy, but they have not reached a unanimous conclusion (DeWall and Baumeister, 2006; Cordaro, 2011; Bass et al., 2014). The present work adopted a neuroscience research method to probe into the temporal dynamics of neural mechanisms behind this phenomenon. The results showed a marginally more positive shift in central N2 amplitudes when watching painful stimuli in contrast with neutral stimuli, regardless of group type. As for the parietal P3 component, painful pictures elicited significantly smaller amplitudes than neutral pictures in the social exclusion group, whereas no ERP responses differed in the social inclusion group. The social exclusion group showed significantly smaller P3 amplitudes than the social inclusion group did while watching painful stimuli. There was a different pattern at the late LPP stage when the LPP amplitudes elicited by painful stimuli were significantly larger than those induced by neutral stimuli in both groups. However, the social exclusion group showed smaller LPP amplitudes than the social inclusion group did while watching painful stimuli.

According to previous empathy studies, N2 is considered to reflect affective sharing or affective arousal (Fan and Han, 2008; Mella et al., 2012). We found that at the central site, painful stimuli elicited marginally significant smaller N2 amplitudes than neutral stimuli did. This finding is consistent with what other ERP studies found (Fan and Han, 2008; Luo et al., 2018a). Meanwhile, as expected, the N2 differences between pain/neutral stimuli did not differ between the two groups, which suggests that acute social pain does not influence an early empathic response toward the pain of strangers. Our results are consistent with a previous study, which suggests that the priming effect of physical pain does not affect the automatic process of pain empathy (Meng et al., 2013).

The P3 component is considered to reflect how people engage attention resources to process and evaluate stimuli (Polich, 2007; Fan and Han, 2008; Decety et al., 2010; Hajcak et al., 2010). Previous studies found that painful images elicited more positive P3 amplitudes than neutral images do, and the pain empathy response is indexed by the differentiation between pain and no-pain (Fan and Han, 2008; Decety et al., 2010; Ikezawa et al., 2013; Coll, 2018a). Studies also suggest that some factors such as state anxiety and medical experience induce the absence of empathic response indexed by the non-significant ERP differentiation between pain and no-pain (Decety et al., 2010; Luo et al., 2018b). However, our results showed that painful pictures even induced smaller P3 amplitudes than neutral pictures did in the social exclusion group. This may suggest that social exclusion group participants not only decreased attention to the pain of others but also increased attention to neutral stimuli. People tend to allocate more attention to threatening stimuli (Bar-Haim et al., 2007; Sharpe et al., 2009; Chan et al., 2010; Cisler and Koster, 2010). Studies have also found that there is another phenomenon, attentional avoidance, which refers to allocating attention to locations opposite to the location of the threat cue (Mogg et al., 2004; Cisler et al., 2009; Cisler and Koster, 2010). Individuals with chronic pain show an attentional pattern of vigilance-avoidance, namely initial vigilance and then subsequent avoidance of negative information (or focus on positive information) (Yang et al., 2013; Priebe et al., 2015; Todd et al., 2015). The threat interpretation model argues that avoidance toward negative stimuli is important in high-threat environments and biases toward positive stimuli can help individuals distract themselves from pain (Todd et al., 2015). Meantime, previous studies suggest that attentional avoidance can be an effective strategy for dealing with anxiety (MacLeod et al., 2009), stress (Wald et al., 2010), and regulating negative emotion (Dunning and Hajack, 2009; Cisler and Koster, 2010). Our behavioral results showed that participants in the social exclusion group suffered social pain, and their basic needs were threatened. Therefore, our results suggest that socially excluded participants may adopt attetional avoidance during the empathic task as a strategy to handle previous social pain and to foster recovery. Physical pain of others is highly related to direct pain. Meta-analyses of fMRI studies have shown that pain empathy activates some similar brain areas as direct physical pain does: the bilateral anterior insular and anterior midcingulate cortex (Fan et al., 2011; Lamm et al., 2011). Loggia et al. (2008) found that when subjects were positively empathizing with the individual in pain, their own pain experience was intensified. Hence, participants in the social exclusion group may feel overwhelmed more easily when trying to empathize with others in pain as they have to suffer two kinds of pain: previous social pain and physical pain they share with others who are in pain situation. In this case, decreasing their attention to the painful stimuli of others and increasing attention to neutral stimuli that are not related to any form of pain may help participants in the social exclusion group avoid suffering more pain and distract themselves from previous social pain. Future study can further verify whether social exclusion induce attentional avoidance toward the pain of others by dot probe paradigm or eye movement technique. This finding has profound significance and important practical value in understanding and helping clients who experience social exclusion, which could also inform effective intervention and prevention efforts.

In social inclusion group, classical ERP effects did not appear in P3 stage, which could be shown by similar P3 amplitudes for painful stimuli and neutral stimuli. This does not support our hypothesis that social inclusion group would show larger P3 amplitudes to painful stimuli compared with neutral stimuli. This unusual phenomenon should be noticed and might indicate that social inclusion weakens pain empathy at P3 stage. However, the social inclusion group displayed significantly larger P3 amplitudes than the social exclusion group did only for painful stimuli. Therefore, our results indicate that painful stimuli still induce empathic responses in the social inclusion group at the P3 stage while participants in the social inclusion group increase attentional level to neutral stimuli. A meta-analysis study on social exclusion studies has shown that social inclusion causes a slight increase in positive affect (Blackhart et al., 2009). Positive affect has proved to increase attention span (Rowe et al., 2006). Combined our results with these findings (Rowe et al., 2006) together, one potential reason may be that social inclusion increases attention span which then increases attention levels to neutral stimuli. Although most of previous studies on Cyberball exclusion adopted social inclusion group as a control group as we did, our results suggest that social inclusion group may not be the most appropriate control group because social inclusion may also affect attention. However, we did not assess the affective state before the manipulation of social exclusion, the meta-analysis can only provide us a potential explanation. Hence, future research can measure the affective state before the Cyberball task and set a new control group of which participants finish the same Cyberball game as participants in the social inclusion group do with the only difference of being informed that they are going to play a Cyberball game with a computer.

LPP is considered to reflect a more controlled processing representing an ongoing positive increase in cognitive resources and being an index of cognitive evaluation (Fan and Han, 2008; Hajcak et al., 2010; Cochran et al., 2017; Meng et al., 2019). LPP is more positive in response to negative stimuli than to neutral stimuli (Cuthbert et al., 2000; Fan and Han, 2008; Coll, 2018b). We found that LPP amplitudes elicited by painful stimuli were significantly larger than those elicited by neutral stimuli regardless of groups. This indicates that the social exclusion group also exhibits empathic response at the late LPP stage. Compared with neutral stimuli, negative stimuli seem to recruit more physiological and psychological resources due to the evaluation of evolutionary importance (Yuan et al., 2007). Following a top-down reappraisal of the painful pictures, combined with the environment and their own experiences participants in the social exclusion group may become more aware of the importance of painful stimuli. Meanwhile, it has been shown that empathy promotes prosocial behavior and is widely appreciated by society (Coke et al., 1978; Batson et al., 2002). They may also regulate the empathic response to conform to social expectations by putting more cognitive efforts into painful stimuli than neutral stimuli. Together, this could explain the reason why the absence of empathic responses at the P3 stage in the social exclusion group reappears at the late LPP stage. However, we also found that the social exclusion group showed smaller LPP amplitudes than the social inclusion group did only for painful stimuli. This indicates that social exclusion impairs individuals' cognitive evaluation ability during the process of empathy and hinders the allocation of cognitive resources to evaluate and process the pain of others compare to social inclusion. This phenomenon could be explained from the perspective of cognition. Late top-down controlled empathy processes need a high level of cognitive control, including self-control and response inhibition (Decety and Lamm, 2006; Mella et al., 2012). The “threat value of pain” hypothesis also argues that the inhibition of the self-protective response, namely the inhibition of escaping from the pain of others, is important during the empathic task (Yamada and Decety, 2009; Decety et al., 2012). Social exclusion has been found to impair self-control (Baumeister et al., 2005; Campbell et al., 2006) and response inhibition (Otten and Jonas, 2012; Xu et al., 2016) while social inclusion has proved to promote self-regulation (DeWall et al., 2008) and cognitive functioning (Shapira et al., 2007). Our behavioral results are consistent with these studies by showing that the need for control scores were significantly smaller in the social exclusion group than in the social inclusion group. Hence, one explanation is that social exclusion impairs individuals' cognitive control ability, which makes it more difficult to reappraise and allocate cognitive resources to the pain of others and inhibits the self-protection response during empathy.

Our behavioral results showed that there was no group differences in subjective unpleasantness ratings regardless of perspectives. This is consistent with the results of a previous study that the social exclusion group and the social inclusion group did not differ in subjective state empathy (Bass et al., 2014). However, we cannot conclude from these findings that social exclusion has no effect on individuals' subsequent pain empathy because our ERP results showed significant differences between groups. ERP is less susceptible to social desirability compared to self-report method (Mostafa, 2014). One possible explanation may be that the self-reported empathy is more likely to be affected by social desirability and hence the differences between groups cannot be directly observed when adopting this method. This finding also demonstrates the necessity to study this phenomenon through more objective electrophysiological methods such as ERP.

Past studies have shown that patients suffering from pain also have abnormal empathic responses to the pain of others. Ma et al. (2020) investigated how chronic low back pain changes empathy and the results showed that patients with the chronic low back pain displayed lower scores on the subscale scores of emotional disconnection and cognitive empathy, and the discomfort rating of watching painful stimuli. Shin et al. (2013) focused on the empathic abilities differences between patients with complex regional pain syndrome (CRPS) Type I and healthy control subjects and found that patients with CRPS showed poorer performance in accuracy of identifying emotional states of others and there was a significant association between the deficit in social-emotion recognition and the affective dimension of pain. Another study has also shown that CRPS patients displayed impaired cognitive and emotional empathic abilities indexed by lower scores in perspective taking and empathic concern and higher scores in personal distress of the IRI (Sohn et al., 2016). Studies on the neural mechanism of clinical chronic pain showed that the chronic low back pain patients displayed multiple abnormal brain pathways centered on the anterior insula (AI) (Ma et al., 2020) and patients with chronic pain disorder displayed lower activation of the left perigenual ACC (Noll-Hussong et al., 2013). AI and ACC have been considered as key areas of pain empathy (Fan et al., 2011; Lamm et al., 2011). These studies suggest that clinical patients with chronic pain have impaired empathy abilities and there also exist some functional abnormalities in brain areas related to empathy. The current study explores how social pain affects subsequent pain empathy by manipulating social exclusion in lab. Our results suggest that individuals suffering social pain have impaired pain empathy at late cognitive controlled stage. Specifically, the social exclusion group displayed attentional avoidance to the pain of others during the process of empathy. They also allocated less attentional and cognitive resources to the pain of others. This indicates that whether an individual suffers from acute social pain or long-term chronic pain, their cognitive processing of the emotions of others and their social cognitive ability are weakened. Empathy plays an essential role in social interaction. Hence the impaired abilities of empathy in patients with chronic pain may affect the social support receiving from family members and the quality of interpersonal relationships (Sohn et al., 2016). Similarly, the reduced pain empathy by social pain may further affect the social interaction and interpersonal relationships of excluded individuals, and increase the possibility of further social exclusion. The enlightenment for future research is that helping socially excluded individuals improve their recognition and understanding of other people's emotions may reduce possible impairment in social cognitive functioning.

In conclusion, the current ERP study provides new neuroscientific insights into how acute social exclusion dynamically affects pain empathy. In comparison with previous studies, the present study provides a new perspective by showing that this effect is a dynamic process. Our study suggests that early sensory processing elicited by the perception of pain during the automatic emotional sharing stage (N2) is not influenced by social pain, but, during late cognitive controlled processing, pain empathy is absent at the P3 stage and is less obvious at the LPP stage. Our findings can also provide an insight into resolving previous inconsistent findings in this field. The current study shows that social exclusion does not affect early affective sharing but that it down-regulates the late controlled processing of the pain of others. Meanwhile, another study adopting the same Cyberball task suggests that social exclusion does not influence pain empathy (Bass et al., 2014). The previous study measured empathy by asking subjects to give subjective reports on state empathy. The current study adopted ERP with high temporal resolution which provides a combination of physiological data and subjective reports, improving the sensitivity and accuracy of the results, and making them relatively more objective (Mostafa, 2014). We assume that the inconsistent results could be attributed to the fact that subjective reports of empathy are more liable to social desirability (Deshields et al., 1995; Logan et al., 2008) and could not obtain accurate dynamic information. One limitation is that we have not investigated the effect of persistent ostracism which limits the generalization and application of the results. For future studies, it is recommended that the influence of previous persistent ostracism should be considering. This would establish an experimental situation with more ecological validity should be considering, resulting in more objective and scientific research results. Another limitation is that we did not measure the affective state before the Cyberball task. Although we randomly allocated participants to either social exclusion group or social inclusion group, we did not take any steps to make sure all participants keep calm before the manipulation. Only measuring affective state after Cyberball may not necessarily reflect how Cyberball exclusion affects emotion. Future studies should assess the affective state both before and after the manipulation of social exclusion. Only with the comparison to the affective state before social exclusion can we obtain how emotions change in the social exclusion group and draw more convincing conclusions.

## Data Availability Statement

The raw data supporting the conclusions of this article will be made available by the authors, without undue reservation.

## Ethics Statement

The studies involving human participants were reviewed and approved by Academic Committee of South China Normal University. The patients/participants provided their written informed consent to participate in this study.

## Author Contributions

MF designed the experiments, analyzed the data, and wrote the manuscript. JJ and XZ designed the experiments and worked on the final version of the manuscript. PL and YP collected and analyzed the data. DX, GY, SZ, and WC collected the data. All authors contributed to the article and approved the submitted version.

## Conflict of Interest

The authors declare that the research was conducted in the absence of any commercial or financial relationships that could be construed as a potential conflict of interest.
